# The Impact of Primary Versus Secondary Muscle-invasive Bladder Cancer at Diagnosis on the Response to Neoadjuvant Chemotherapy

**DOI:** 10.1016/j.euros.2022.05.001

**Published:** 2022-05-28

**Authors:** David D'Andrea, Shahrokh F. Shariat, Francesco Soria, Andrea Mari, Laura S. Mertens, Ettore Di Trapani, Diego M. Carrion, Benjamin Pradere, Renate Pichler, Ronan Filippot, Guillaume Grisay, Francesco Del Giudice, Ekaterina Laukhtina, David Paulnsteiner, Wojciech Krajewski, Sonia Vallet, Martina Maggi, Ettore De Berardinis, Mario Álvarez-Maestro, Stephan Brönimann, Fabrizio Di Maida, Bas W.G. van Rhijn, Kees Hendricksen, Marco Moschini, European Association of Urology Young Academic Urologists Urothelial Carcinoma Working Group

**Affiliations:** a Department of Urology, Medical University of Vienna, Vienna, Austria; b Department of Urology, University of Texas Southwestern Medical Center, Dallas, TX, USA; c Department of Urology, Weill Cornell Medical College, New York, NY, USA; d Institute for Urology and Reproductive Health, I.M. Sechenov First Moscow State Medical University, Moscow, Russia; e Department of Urology, Second Faculty of Medicine, Charles University, Prague, Czech Republic; f Hourani Center for Applied Scientific Research, Al-Ahliyya Amman University, Amman, Jordan; g Department of Urology, AOU Città della Salute e della Scienza, Torino School of Medicine, Turin, Italy; h Department of Experimental and Clinical Medicine, University of Florence, Oncologic Minimally Invasive Urology and Andrology Unit, Careggi Hospital, Florence, Italy; i Department of Urology, The Netherlands Cancer Institute-Antoni van Leeuwenhoek Hospital, Amsterdam, The Netherlands; j Department of Urology, European Institute of Oncology IRCCS, Milan, Italy; k Department of Urology, Torrejon University Hospital, Madrid, Spain; l Francisco de Vitoria University, Madrid, Spain; m Department of Urology, Medical University Innsbruck, Austria; n Department of Cancer Medicine, Institut Gustave Roussy, Villejuif, France; o Department of Maternal Infant and Urologic Sciences, Sapienza University of Rome, Policlinico Umberto I Hospital, Rome, Italy; p Department of Minimally Invasive and Robotic Urology, University Center of Excellence in Urology, Wrocław Medical University, Wrocław, Poland; q Department of Internal Medicine II, University Hospital Krems, Karl Landsteiner University of Health Sciences, Krems an der Donau, Austria; r Department of Urology, Hospital Universitario La Paz, Madrid, Spain; s Department of Urology, IRCCS Ospedale San Raffaele and Vita-Salute San Raffaele University, Milan, Italy

**Keywords:** Neoadjuvant chemotherapy, Bladder cancer, Response, Survival, Primary, Secondary

## Abstract

**Background:**

There might be differential sensitivity to neoadjuvant chemotherapy (NAC) in patients with primary muscle-invasive bladder cancer (MIBC) in comparison to patients with secondary MIBC after a history of non–muscle-invasive disease.

**Objective:**

To investigate pathologic response rates and survival associated with primary versus secondary MIBC among patients treated with cisplatin-based NAC for cT2–4N0M0 MIBC.

**Design, setting, and participants:**

Oncologic outcomes were compared for 350 patients with primary MIBC and 64 with secondary MIBC treated with NAC and radical cystectomy between 1992 and 2021 at 11 academic centers. Genomic analyses were performed for 476 patients from the Memorial Sloan Kettering/The Cancer Genome Atlas cohort.

**Outcome measurements and statistical analysis:**

The outcome measures were pathologic objective response (pOR; ≤ypT1 N0), pathologic complete response (pCR; ypT0 N0), overall mortality, and cancer-specific mortality.

**Results and limitations:**

The primary MIBC group had higher pOR (51% vs 34%; *p* = 0.02) and pCR (33% vs 17%; *p* = 0.01) rates in comparison to the secondary MIBC group. On multivariable logistic regression analysis, primary MIBC was independently associated with both pOR (odds ratio [OR] 0.49, 95% confidence interval [CI] 0.26–0.87; *p* = 0.02) and pCR (OR 0.41, 95% CI 0.19–0.82; *p* = 0.02). However, on multivariable Cox regression analysis, primary MIBC was not associated with overall mortality (hazard ratio 1.70, 95% CI 0.84–3.44; *p* = 0.14) or cancer-specific mortality (hazard ratio 1.50, 95% CI 0.66–3.40; *p* = 0.3). Genomic analyses revealed a significantly higher *ERCC2* mutation rate in primary MIBC than in secondary MIBC (12.4% vs 1.3%; *p* < 0.001).

**Conclusions:**

Patients with primary MIBC have better pathologic response rates to NAC in comparison to patients with secondary MIBC. Chemoresistance might be related to the different genomic profile of primary versus secondary MIBC.

**Patient summary:**

We investigated the treatment response to neoadjuvant chemotherapy (NAC; chemotherapy received before the primary course of treatment) and survival for patients with a primary diagnosis of muscle-invasive bladder cancer (MIBC) in comparison to patients with a history of non–muscle-invasive bladder cancer that progressed to MIBC. Patients with primary MIBC had a better response to NAC but this did not translate to better survival after accounting for other tumor characteristics.

## Take Home Message

Our study generates the hypothesis that patients with a primary diagnosis of muscle-invasive bladder cancer (MIBC) might have a better pathologic response to cisplatin-based neoadjuvant chemotherapy in comparison to patients with a history of non–muscle-invasive bladder cancer that progressed to MIBC.

## 1 Introduction

Neoadjuvant chemotherapy (NAC) with a cisplatin-based combination followed by radical cystectomy (RC) is the standard of care for patients with cT2–4N0M0 muscle-invasive bladder cancer (MIBC) who are cisplatin-eligible [1]. Although current evidence supports an overall improvement in survival, not every patient will benefit from NAC [2], [3]. Patient selection is of paramount importance in order to identify those who will not respond to NAC to avoid NAC toxicity and a delay to definitive therapy. To address this issue, several working groups have investigated the predictive role of clinical, pathologic, and molecular characteristics [3], [4], [5], [6], [7]. In this context, retrospective series have shown that non–muscle-invasive bladder cancer (NMIBC) progressing to MIBC (secondary MIBC) might have differential oncologic outcomes in comparison to primary MIBC [8]. However, the role of tumor status, especially in patients treated with NAC, has not been fully elucidated [8], [9], [10]. To fill this gap in knowledge, we investigated the association of primary versus secondary MIBC with pathologic response to NAC and survival using data from an international collaborative group.

## 2 Patients and methods

### 2.1 Patient characteristics

We reviewed our multi-institutional database to identify 1002 patients treated with NAC and RC at 11 academic centers between 1992 and 2021. Only patients with cT2–4N0M0 MIBC treated with three or four cycles of cisplatin-based combination NAC were included. Patients with secondary MIBC who did not receive a second-look transurethral resection of bladder tumor (TURB) at first diagnosis to confirm NMIBC status were excluded. The patient inclusion/exclusion process is shown in Supplementary Figure 1. Primary MIBC was defined as invasion into or beyond the muscularis propria on either initial or second-look TURB. Secondary MIBC was defined as MIBC occurring after an initial diagnosis of ≤T1 NMIBC confirmed at second-look TURB. Chemotherapy cycles consisted of gemcitabine-cisplatin or dose-dense methotrexate-vinblastine-doxorubicin hydrochloride-cisplatin. Other NAC regimens included cisplatin-methotrexate-vinblastine, gemcitabine-cisplatin-paclitaxel, cisplatin-5-fluorouracil, cisplatin-farmorubicin, and cisplatin-etoposide. None of the patients was treated with split-dose NAC.

### 2.2 Endpoints

The primary endpoint of the study was the association of tumor status (primary vs secondary) with pathologic objective response (pOR), defined as stage ≤ypT1N0 at RC after NAC [11]. Secondary endpoints were the association of tumor status with pathologic complete response (pCR), overall mortality (OM), and cancer-specific mortality (CSM).

### 2.3 Genomic analysis

We used publicly available whole-exome or targeted sequencing data for 476 patients with MIBC to investigate a possible relationship between genomic mutations and tumor status. The cohort included 334 patients analyzed via whole-exome sequencing and 142 patients analyzed via Memorial Sloan Kettering (MSK)-IMPACT sequencing. Sequencing was performed on fresh frozen or formalin-fixed paraffin-embedded specimens obtained via transurethral resection or RC. All patients were chemotherapy-naïve. We investigated a panel of genes on the basis of prior reports, current ongoing prospective trials, and genes found to have the highest mutation rates in the MSK/The Cancer Genome Atlas (TCGA) cohort [2], [9], [12], [13], [14]. All data used for this analysis are available at https://cbioportal.org.

### 2.4 Statistical analyses

Point estimates and 95% confidence intervals (CIs) for pathologic response were generated using exact binomial distributions. The association of tumor status with pOR and pCR was investigated using univariable and multivariable logistic regression analyses. The association of tumor status with OM and CSM was investigated using univariable and multivariable Cox regression analyses. The multivariable models were adjusted for clinicopathologic characteristics known to be associated with the outcomes investigated. Survival was plotted using the Kaplan-Meier method and compared using the log-rank test. The discrimination of the multivariable models was assessed using the Harrel c index. The frequency of somatic genomic mutations was compared between groups using Fisher’s exact test. All tests were two-sided and statistical significance was set at *p* < 0.05. We used R (R Foundation for Statistical Computing, Vienna, Austria) for statistical analyses.

## 3 Results

Overall, 350 patients (85%) had primary MIBC and 64 (15%) had secondary MIBC. pOR was achieved in 190 patients (46%; 95% CI 41–51%) and pCR in 124 patients (30%; 95% CI 26–35%) overall. Patients with primary MIBC had higher pOR (49% vs 31%; p = 0.01) and pCR (33% vs 16%; *p* = 0.007) rates, were younger (64 vs 68 yr; *p* = 0.01), and had more advanced disease at RC in comparison to patients with secondary MIBC. There were no significant differences between the groups for other clinicopathologic characteristics (Table 1).

**Table 1:** Clinicopathologic characteristics of 414 patients treated with cisplatin-based NAC for cT2-4N0M0 bladder cancer, stratified by tumor status

Variable	Overall	MIBC status		*p* value^a^
	(*n* = 414)	Primary	Secondary	
		(*n* = 350)	(*n* = 64)	
Median age, yr (IQR)	64 (57–70)	64 (57–69)	68 (60–73)	0.015
Sex, *n* (%)				0.20
Female	104 (25)	92 (26)	12 (19)	
Male	310 (75)	258 (74)	52 (81)	
cT stage, *n* (%)				0.81
T2	280 (68)	236 (67)	44 (69)	
T3	95 (23)	82 (23)	13 (20)	
T4	39 (9.4)	32 (9.1)	7 (11)	
ypT stage, *n* (%)				0.10
T0	132 (32)	119 (34)	13 (20)	
T1/Ta/Tis	70 (17)	60 (17)	10 (16)	
T2	83 (20)	69 (20)	14 (22)	
T3/T4	129 (31)	102 (29)	27 (42)	
ypN stage, *n* (%)				0.053
N0	319 (77)	277 (79)	42 (66)	
N1	38 (9.2)	29 (8.3)	9 (14)	
N2	42 (10)	30 (8.6)	12 (19)	
N3	11 (2.7)	10 (2.9)	1 (1.6)	
Nx	4 (1.0)	4 (1.1)	0 (0)	
Median lymph nodes removed, *n* (IQR)	20 (14–26)	19 (14–26)	21 (15–25)	0.35
Lymphovascular invasion, *n* (%)	61 (21)	45 (19)	16 (30)	0.068
Not reported	123	112	11	
Concomitant carcinoma in situ, *n* (%)	71 (17)	56 (16)	15 (24)	0.15
Not reported	7	6	1	
STSM, *n* (%)				0.61
Negative	378 (91)	321 (92)	57 (89)	
Positive	22 (5.3)	18 (5.1)	4 (6.2)	
Not evaluable	14 (3.4)	11 (3.1)	3 (4.7)	
Variant histology, *n* (%)				0.29
Absent	370 (92)	315 (93)	55 (89)	
Present	30 (7.5)	23 (6.8)	7 (11)	
Not reported	14	12	2	
NAC cycles, *n* (%)				0.49
3 cycles	127 (31)	105 (30)	22 (34)	
4 cycles	287 (69)	245 (70)	42 (66)	
NAC regimen, *n* (%)				0.34
ddMVAC	77 (19)	69 (20)	8 (12)	
Gemcitabine-cisplatin	324 (78)	269 (77)	55 (86)	
Other	13 (3.1)	12 (3.4)	1 (1.6)	

ddMVAC = dose-dense methotrexate-vinblastine-doxorubicin hydrochloride-cisplatin; IQR = interquartile range; MIBC = muscle-invasive bladder cancer; NAC = neoadjuvant chemotherapy; STSM = soft-tissue surgical margin.

a Wilcoxon rank-sum test, Pearson’s χ^2^ test, or Fisher’s exact test, as appropriate.

On univariable logistic regression analysis, patients with primary MIBC were more likely to experience pOR (odds ratio [OR] 0.48, 95% CI 0.27–0.84; *p* = 0.012) and pCR (OR 0.38, 95% CI 0.18–0.75; *p* = 0.008). On multivariable logistic regression analyses adjusted for the effect of patient sex, age, clinical stage, number of NAC cycles, and NAC regimen administered, tumor status remained significantly associated with pOR (OR 0.47, 95% CI 0.26–0.83; *p* = 0.011) and pCR (OR 0.39, 95% CI 0.18–0.78; *p* = 0.011; Table 2).

**Table 2:** Multivariable logistic regression analyses of the association of tumor status with pathologic objective response (≤ypT1N0) and pathologic complete response (ypT0N0) among 414 patients treated with NAC and radical cystectomy for cT2–4N0M0 bladder cancer

Variable	Pathologic objective response		Pathologic complete response	
	OR (95% CI)	*p* value	OR (95% CI)	*p* value
Tumor status (secondary vs primary)	0.47 (0.26–0.83)	0.011	0.39 (0.18–0.78)	0.011
Age in years	1.00 (0.98–1.02)	>0.9	0.99 (0.97–1.02)	0.5
Male sex	1.16 (0.73–1.83)	0.5	0.83 (0.51–1.37)	0.5
cT stage				
T2	Reference		Reference	
T3	0.52 (0.31–0.86)	0.012	0.53 (0.29–0.92)	0.027
T4	0.46 (0.22–0.95)	0.039	0.39 (0.15–0.90)	0.037
NAC cycles (4 vs 3)	1.35 (0.86–2.13)	0.2	1.32 (0.81–2.17)	0.3
NAC regimen				
ddMVAC	Reference		Reference	
Gemcitabine-cisplatin	1.16 (0.69–1.96)	0.6	1.10 (0.63–1.98)	0.7
Other	1.71 (0.51–6.28)	0.4	1.75 (0.50–6.03)	0.4
Harrell’s c index		0.62		0.62

NAC = neoadjuvant chemotherapy; ddMVAC = dose-dense methotrexate-vinblastine-doxorubicin hydrochloride-cisplatin; OR = odds ratio; CI = confidence interval.

**Fig. 1:**
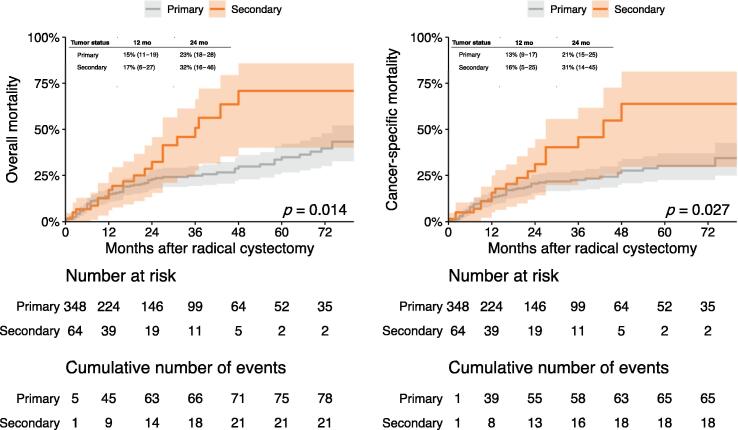
Cumulative incidence curves for overall mortality and cancer-specific mortality among 412 patients treated with cisplatin-based combination neoadjuvant chemotherapy and radical cystectomy for cT2–4N0M0 bladder cancer.

During median follow-up of 19 mo (interquartile range 9–40) for surviving patients, 102 patients died, of whom 86 died of bladder cancer. Figure 1 shows cumulative incidence curves for OM and CSM. On univariable Cox regression analysis, secondary MIBC was associated with worse OM (hazard ratio [HR] 1.81, 95% CI 1.12–2.94; *p* = 0.02) and CSM (HR 1.79, 95% CI 1.06–3.02; *p* = 0.03). However, tumor status was no longer associated with survival outcomes on multivariable Cox regression analysis (Table 3).

**Table 3:** Multivariable Cox regression analyses investigating the association of tumor status with overall mortality and cancer-specific mortality among 412 patients treated with neoadjuvant chemotherapy and radical cystectomy for cT2–4N0M0 bladder cancer

Variable	Overall mortality		Cancer-specific mortality	
	HR (95% CI)	*p* value	HR (95% CI)	*p* value
Tumor status (secondary vs primary)	1.49 (0.79–2.84)	0.2	1.43 (0.69–2.93)	0.3
Age in years	1.00(0.97–1.03)	>0.9	0.99 (0.96–1.02)	0.6
Male sex	0.64 (0.35–1.17)	0.15	0.50 (0.25–0.99)	0.048
NAC cycles (4 vs 3)	0.91 (0.50–1.66)	0.8	0.63 (0.32–1.24)	0.2
NAC regimen				
ddMVAC	Reference		Reference	
Gemcitabine-cisplatin	0.81 (0.42–1.56)	0.5	0.64 (0.31–1.33)	0.2
Other	1.34 (0.18–10.1)	0.8	2.32 (0.25–21.5)	0.5
ypT stage				
T0	Reference		Reference	
T1/Ta/Tis	1.63 (0.46–5.74)	0.4	9.05 (1.03–79.4)	0.047
T2	1.88 (0.59–5.97)	0.3	4.49 (0.50–40.8)	0.2
T3/T4	5.07 (1.79–14.3)	0.002	19.4 (2.43–155)	0.005
ypN stage				
N0	Reference		Reference	
N1	2.42 (1.10–5.35)	0.029	3.37 (1.42–7.99)	0.006
N2	3.17 (1.39–7.24)	0.006	4.16 (1.64–10.5)	0.003
N3	2.02 (0.51–8.04)	0.3	3.42 (0.81–14.5)	0.10
Nx	0.90 (0.04–19.9)	>0.9	0.65 (0.02–17.3)	0.8
Lymphovascular invasion	0.92 (0.46–1.83)	0.8	0.82 (0.39–1.75)	0.6
Concomitant carcinoma in situ	0.88 (0.43–1.80)	0.7	1.31 (0.60–2.85)	0.5
Soft-tissue surgical margin				
Negative	Reference		Reference	
Positive	1.00 (0.33–3.06)	>0.9	1.40 (0.44–4.49)	0.6
Not evaluable	0.41 (0.06–2.67)	0.4	0.42 (0.05–3.41)	0.4
Harrell’s c index		0.78		0.81

NAC = neoadjuvant chemotherapy; ddMVAC = dose-dense methotrexate-vinblastine-doxorubicin hydrochloride-cisplatin; HR = hazard ratio; CI = confidence interval.

**Fig. 2:**
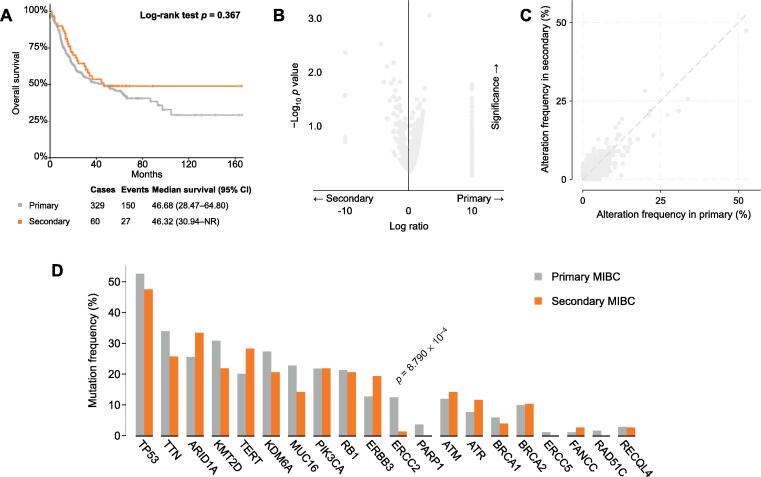
Genomic analyses for 476 patients with muscle-invasive bladder cancer stratified by tumor status. (A) Kaplan-Meier curves comparing overall survival for patients with primary and secondary MIBC. (B) Volcano plot and (C) scatter plot of the association of gene mutations with primary and secondary MIBC. (D) Gene alteration frequency stratified by tumor status.

In the cohort used for genomic analysis, 78 patients had secondary MIBC and 398 had primary MIBC. Overall, the number of gene mutations was comparable between the two cohorts (Fig. 2B, C). We found a significantly higher *ERCC2* mutation rate in primary MIBC than in secondary MIBC (12.4% vs 1.3%; *p* < 0.001; Fig. 2D). There was no significant difference in overall survival between the primary and secondary MIBC groups (*p* = 0.4; Fig. 2A).

## 4 Discussion

We investigated differential oncologic outcomes for patients with primary and secondary MIBC. We found a significant association between tumor status and pathologic outcomes. Our findings have several significant implications for clinical decision-making and patient counseling. While NAC is recommended for all patients with cT2–4N0 MIBC, not every patient is likely to respond to this therapy [15]. Therefore, tools for patient selection are needed in daily practice. Molecular subtyping and biomarkers might help clinicians in the near future [6], [16]. However, until their clinical impact and cost effectiveness are evaluated in prospective trials, physicians must rely on clinical and pathologic characteristics and patient comorbidities in the decision-making process [3], [5], [17], [18]. Our study adds to the relevant information that might help during this process. We showed that tumor status might be associated with NAC response and this could help in patient selection for upfront RC if confirmed in prospective trials.

Several studies have investigated the association of tumor status with oncologic outcomes, with conflicting results [10], [19], [20], [21], [22]. However, these studies did not assess the effect of tumor status in patients treated with NAC. A recent systematic review and meta-analysis found similar 5-yr and 10-yr overall survival and cancer-specific survival (CSS) rates between primary MIBC and secondary MIBC [8]. Interestingly, in subgroup analyses of patients treated with NAC, those with secondary MIBC had worse 5-yr CSS (HR 1.5; *p* = 0.04) but not 10-year CSS. These findings generate the hypothesis that there might be a differential response to NAC according to tumor status and that worse survival might be attributable to a delay in RC. However, these data are from a small number of retrospective single-center studies with a limited number of patients. Moreover, these series did not report on pathologic response rates to NAC [17], [23], [24].

We found that patients with secondary MIBC had higher rates of non–organ-confined disease (stage ypT3/4 and/or N+) at RC, were less likely to respond to NAC, and had shorter survival in comparison to patients with primary MIBC. Our findings are in accordance with results reported for a retrospective series of 288 patients [9]. The authors hypothesized that one of the reasons for this detrimental effect could be clonal cell selection resulting from previous intravesical chemotherapies and bacillus Calmette-Guérin immunotherapy.

Alterations in DNA damage repair genes have been associated with chemosensitivity to cisplatin-based NAC [12], [13], [14]. Therefore, we investigated genomic differences between primary and secondary MIBC as a possible rationale to explain the differential response. Our genomic analysis of the MSK/TCGA cohort showed that the *ERCC2* mutation rate was higher in primary MIBC than in secondary MIBC. Somatic missense mutations in *ERCC2* have emerged as clinically significant biomarkers for chemotherapy response in bladder cancer in several trials [13], [14], [25]. Our study confirms the findings of a previous report showing a significantly higher rate of *ERCC2* mutation in primary MIBC [9] and reinforces the theory that this might correlate with better chemosensitivity and therefore better pathologic response and survival.

Currently, the majority of the evidence shows that for patients treated with RC alone, tumor status is not associated with oncologic outcomes [8]. However, our analysis generates the hypothesis that this might not to be true for patients treated with NAC and RC. Chemoresistance related to disparate genomic characteristics between primary and secondary MIBC might lead to a delay in RC, disease progression, and therefore worse oncologic outcomes. This could partly explain our findings of worse pathologic responses and survival for patients treated with NAC but comparable survival in the MSK/TCGA cohort of patients treated with RC only.

There are several limitations to our study that should be considered. We acknowledge the selection bias inherent to the retrospective design and the lack of a control cohort of patients treated with RC only. We could not account for patient performance status, comorbidities, surgical quality, and other nonmeasurable confounders. We had no granular information on previous intravesical therapies in the secondary MIBC cohort. There was no central pathology review of the specimens. The preoperative staging and follow-up were not standardized but were based on guidelines [1] and institutional protocols. The median follow-up in our cohort was relatively short and this might have limited the ability to detect a significant difference in multivariable survival analyses. Finally, we acknowledge the long study period resulting in cohort heterogeneity and different treatment protocols between centers over the years.

Despite all the limitations, our findings could serve as rationale for patient counseling in clinical practice and a basis for planning prospective trials with the aim of improving patient selection for NAC.

## 5 Conclusions

Our study generates the hypothesis that patients with secondary MIBC are less likely to respond to NAC in comparison to patients with primary MIBC. This might be related to genomically driven chemoresistance mechanisms that have emerged over the tumor natural history from NMIBC to MIBC, partly as a result of therapy-related clonal selection. These findings suggest that tumor status could be applied to prospectively guide therapy decisions regarding NAC versus immediate RC. However, further evaluation in prospective trials is warranted.

  ***Author contributions***: David D’Andrea had full access to all the data in the study and takes responsibility for the integrity of the data and the accuracy of the data analysis.

*Study concept and design*: D’Andrea, Shariat, Soria, Moschini.

*Acquisition of data*: D’Andrea, Shariat, Soria, Mari, Mertens, Di Trapani, Carrion, Pradere, Pichler, Filippot, Grisay, Del Giudice, Laukhtina, Paulnsteiner, Krajewski, Vallet, Maggi, De Berardinis, Álvarez-Maestro, Brönimann, Di Maida, van Rhijn, Hendricken.

*Analysis and interpretation of data*: D’Andrea, Shariat.

*Drafting of the manuscript*: D’Andrea.

*Critical revision of the manuscript for important intellectual content*: Shariat, Soria, Mari, Mertens, Di Trapani, Carrion, Pradere, Pichler, Filippot, Grisay, Del Giudice, Laukhtina, Paulnsteiner, Krajewski, Vallet, Maggi, De Berardinis, Álvarez-Maestro, Brönimann, Di Maida, van Rhijn, Hendricken, Moschini.

*Statistical analysis*: D’Andrea.

*Obtaining funding*: None.

*Administrative, technical, or material support*: None.

*Supervision*: Shariat, Moschini.

*Other*: None.

  ***Financial disclosures:*** David D’Andrea certifies that all conflicts of interest, including specific financial interests and relationships and affiliations relevant to the subject matter or materials discussed in the manuscript (eg, employment/affiliation, grants or funding, consultancies, honoraria, stock ownership or options, expert testimony, royalties, or patents filed, received, or pending), are the following: None.

  ***Funding/Support and role of the sponsor*:** None.

## References

[1] Witjes J.A., Bruins H.M., Cathomas R. (2021). European Association of Urology guidelines on muscle-invasive and metastatic bladder cancer: summary of the 2020 guidelines. Eur Urol.

[2] D’Andrea D., Black P.C., Zargar H. (2021). Association of age with response to preoperative chemotherapy in patients with muscle-invasive bladder cancer. World J Urol.

[3] D’Andrea D., Black P.C., Zargar H. (2020). Impact of sex on response to neoadjuvant chemotherapy in patients with bladder cancer. Urol Oncol.

[4] Stangl-Kremser M.A., D’Andrea D. (2018). Sarcopenia as a predictive factor for response to upfront cisplatin-based chemotherapy in patients with muscle-invasive urothelial bladder cancer. Urol Int.

[5] Gild P., Vetterlein M.W., Seiler R. (2020). The association of cigarette smoking and pathological response to neoadjuvant platinum-based chemotherapy in patients undergoing treatment for urinary bladder cancer — a prospective European multicenter observational study of the EAU Young Academic Urologists (YAU) Urothelial Carcinoma Working Group. Surg Oncol.

[6] Seiler R., Ashab H.A.D., Erho N. (2017). Impact of molecular subtypes in muscle-invasive bladder cancer on predicting response and survival after neoadjuvant chemotherapy. Eur Urol.

[7] Choi W., Porten S., Kim S. (2014). Identification of distinct basal and luminal subtypes of muscle-invasive bladder cancer with different sensitivities to frontline chemotherapy. Cancer Cell.

[8] Pones M., D’Andrea D., Mori K. (2021). Differential prognosis and response of denovo vs. secondary muscle-invasive bladder cancer: an updated systematic review and meta-analysis. Cancers.

[9] Pietzak E.J., Zabor E.C., Bagrodia A. (2018). Genomic differences between “primary” and “secondary” muscle-invasive bladder cancer as a basis for disparate outcomes to cisplatin-based neoadjuvant chemotherapy. Eur Urol.

[10] Moschini M., Sharma V., Dell’oglio P. (2016). Comparing long-term outcomes of primary and progressive carcinoma invading bladder muscle after radical cystectomy. BJU Int.

[11] Zargar H., Zargar-Shoshtari K., Lotan Y. (2016). Final pathological stage after neoadjuvant chemotherapy and radical cystectomy for bladder cancer—does pT0 predict better survival than pTa/Tis/T1?. J Urol.

[12] Plimack E.R., Dunbrack R.L., Brennan T.A. (2015). Defects in DNA repair genes predict response to neoadjuvant cisplatin-based chemotherapy in muscle-invasive bladder cancer. Eur Urol.

[13] Allen E.M.V., Mouw K.W., Kim P. (2014). Somatic ERCC2 mutations correlate with cisplatin sensitivity in muscle-invasive urothelial carcinoma. Cancer Discov.

[14] Liu D., Plimack E.R., Hoffman-Censits J. (2016). Clinical validation of chemotherapy response biomarker ERCC2 in muscle-invasive urothelial bladder carcinoma. JAMA Oncol.

[15] Yin M., Joshi M., Meijer R.P. (2016). Neoadjuvant chemotherapy for muscle-invasive bladder cancer: a systematic review and two-step meta-analysis. Oncol.

[16] Soria F., Krabbe L.-M., Todenhöfer T. (2019). Molecular markers in bladder cancer. World J Urol.

[17] Moschini M., D’Andrea D., Korn S. (2017). Characteristics and clinical significance of histological variants of bladder cancer. Nat Rev Urol.

[18] Soria F., Black P.C., Fairey A.S. (2021). Neoadjuvant chemotherapy plus radical cystectomy versus radical cystectomy alone in clinical T2 bladder cancer without hydronephrosis. BJU Int.

[19] de Vries R.R., Nieuwenhuijzen J.A., Vincent A., van Tinteren H., Horenblas S. (2010). Survival after cystectomy for invasive bladder cancer. Eur J Surg Oncol.

[20] Breau R.H., Karnes R.J., Farmer S.A. (2014). Progression to detrusor muscle invasion during urothelial carcinoma surveillance is associated with poor prognosis. BJU Int.

[21] Schrier B.P., Hollander M.P., van Rhijn B.W.G., Kiemeney L.A.L.M., Witjes J.A. (2004). Prognosis of muscle-invasive bladder cancer: difference between primary and progressive tumours and implications for therapy. Eur Urol.

[22] Kotb A.F., Kovac E., Kassouf W. (2012). Radical cystectomy for clinically muscle invasive bladder cancer: does prior non-invasive disease affect clinical outcomes?. World J Urol.

[23] Kayama E., Kikuchi E., Fukumoto K. (2018). History of non–muscle-invasive bladder cancer may have a worse prognostic impact in cT2–4aN0M0 bladder cancer patients treated with radical cystectomy. Clin Genitourin Cancer.

[24] Faba O.R., Palou J., Rosales A. (2011). Clinical predictive factors of poor outcome in patients with stage pT0 disease at radical cystectomy. J Urol.

[25] Li Q., Damish A., Frazier Z.J. (2019). ERCC2 helicase domain mutations confer nucleotide excision repair deficiency and drive cisplatin sensitivity in muscle-invasive bladder cancer. Clin Cancer Res.

