# Supplementary material for: The Impact of Primary Versus Secondary Muscle-invasive Bladder Cancer at Diagnosis on the Response to Neoadjuvant Chemotherapy
Source: Eur Urol Open Sci. 2022 May 28;41:74–80. doi: 10.1016/j.euros.2022.05.001 (PMC9257642; doi:10.1016/j.euros.2022.05.001)
Supplement: Supplementary data 1 [file mmc1.docx]

**Supplementary Figure 1**

Patient selection process
